# Male Germ Cell Apoptosis and Epigenetic Histone Modification Induced by *Tripterygium wilfordii* Hook F

**DOI:** 10.1371/journal.pone.0020751

**Published:** 2011-06-15

**Authors:** Ji Xiong, Hu Wang, Guangming Guo, Shuzeng Wang, Liqun He, Haifeng Chen, Ji Wu

**Affiliations:** 1 Key Laboratory for the Genetics of Developmental & Neuropsychiatric Disorders (Ministry of Education), Bio-X Research Institute, Shanghai Jiao Tong University, Shanghai, China; 2 Department of Biological Science and Biotechnology, School of Life Sciences and Biotechnology, Shanghai Jiao Tong University, Shanghai, China; 3 Department of Bioinformatics and Biostatistics, School of Life Sciences and Biotechnology, Shanghai Jiao Tong University, Shanghai, China; 4 Department of Nephrology, Key Laboratory of Liver and Kidney Diseases (Ministry of Education), Shanghai Shuguang Hospital Affiliated with Shanghai University of Traditional Chinese Medicine, Shanghai, China; 5 E-Institute of Traditional Chinese Internal Medicine of Shanghai Municipal Education Commission, Shanghai Traditional Chinese Medicine University, Shanghai, China; Baylor College of Medicine, United States of America

## Abstract

Multiglycosides of *Tripterygium wilfordii* Hook f (GTW), a Chinese herb-derived medicine used as a remedy for rheumatoid arthritis, are considered to be a reversible anti-fertility drug affecting the mammalian spermatids. However, the mechanism behind this effect is still unknown. To study the possible mechanism behind the impact of GTW on spermatogenesis, we administered 4 groups of 4-week-old male mice with different doses of GTW. We found a dose-dependent decrease in the number of germ cells after 40 days of GTW treatment, and an increase in apoptotic cells from the low-dose to the high-dose group. During this same period the dimethylated level of histone H3 lysine 9 (H3K9me2) in GTW-treated testes germ cells declined. Additionally, spermatogonial stem cells (SSCs) from 6-day-old mice were isolated to evaluate the possible effect of GTW or triptolide on development of SSCs. We found a significantly higher incidence of apoptosis and lower dimethylation level of H3K9me2 in the SSCs of GTW or triptolide treatment than in controls. Thus, these data suggest that the GTW-induced apoptosis might be responsible for the fertility impairment in mice. This damage could be traced back to the early stages of spermatogenesis. GTW also affected the epigenetic modification of H3K9 in spermatogenesis. Molecular dynamics simulation suggested that triptolide and dimethylated or trimethylated H3K9 might have similar interaction mechanisms with EED (embryonic ectoderm development). These candidate activation mechanisms provide the first glimpse into the pathway of GTW-induced gonad toxicity, which is crucial for further research and clinical application.

## Introduction

A key physiological process that determines the reproductive ability of adult males is called spermatogenesis, the process by which spermatogonial stem cells (SSCs) develop into mature spermatozoa, also known as sperm cells. Production of normal germ cells throughout this complex process is pivotal to male fertility. Three distinct phases, mitosis, meiosis and spermiogenesis, are involved. SSCs, which reside in the basal layer of the seminiferous tubules of the testis, have the capacity of self-renewal, and first differentiate into primary spermatocytes. After sequential steps of meiotic division, spermatids with haploid chromosomes are formed and finally undergo spermiogenesis to generate mature spermatozoa [1]–[6]. The whole process is regulated by both extrinsic stimuli and intrinsic gene expression, with a series of specific events involved, such as paternal imprinting [7], [8] and meiotic sex chromosome inactivation [9]. Impairment of this well-orchestrated process, either in the germ line cell itself or in the Sertoli cells that support their growth, may lead to male infertility.

Apoptosis is another important regulatory event associated with spermatogenic cell maturation. Huckins found that the degeneration of type A spermatogonia is a common occurrence in rats [10], and apoptotic spermatocytes were observed mostly in stages I, II, VII–IX, XII and XIV. This cell death mechanism is important for the normal development of spermatozoa, as it is one of the key approaches to clearing superfluous or abnormal germ cells. In addition, physiological apoptosis can keep an optimal balance between the numbers of spermatogenic cells and Sertoli cells. This ratio ensures sufficient nutrient support for germ cells from their niches [11]. Pathological apoptosis may result in abnormal spermatogenesis, which could impair the reproductive function.

The process of germ cell development is also controlled by epigenetic mechanisms, including DNA methylation, histone modification and chromatin remodeling. In this study, we focused on the regulation of the lysine 9 dimethylation of histone H3 (H3K9), which is a critical epigenetic marker for gene silencing or repression, and plays an essential role in spermatogenesis. Previous studies have shown that there are three different methylation states on H3K9: mono-, di-, and trimethylation (H3K9me1/me2/me3). H3K9me1 is found almost exclusively during the first half of the S phase, and H3K9me3 is replicated primarily in the mid- to late-S phase. H3K9me2 is replicated throughout most of the S phase [12]. During the development of spermatozoa, dimethylated H3K9 has been observed to concentrate in the X and Y chromosomes at the early stage of spermatogenesis [13], which is deemed to participate in X and Y inactivation.

**Figure 1:**
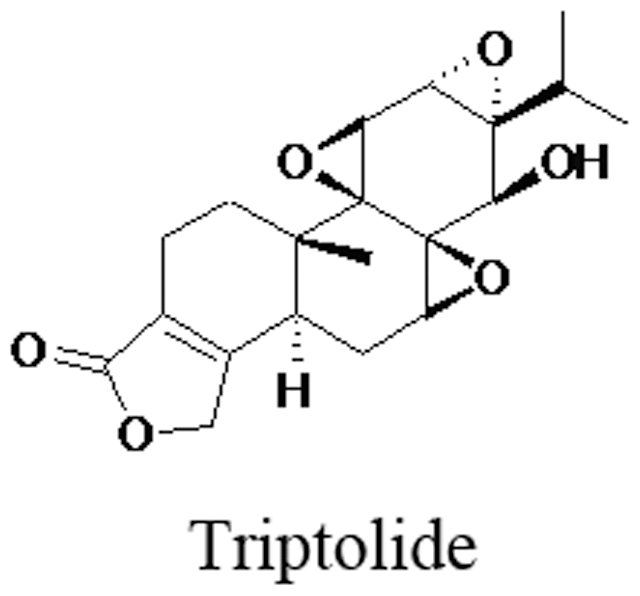
The structure of tripterygium glycoside.

Multiglycosides of *Tripterygium wilfordii* Hook f (GTW), which are isolated from the root xylem of *Tripterygium wilfordii* [14], are used for treating rheumatoid arthritis and various skin disorders. Moreover, Triptolide (Figure 1) is a potent, biologically active compound isolated from Tripterygium wilfordii Hook F. Clinical observation showed a decreased number as well as a reduced vitality of spermatozoa in GTW-treated patients [15]. This result was confirmed in a mouse model [16]. In addition, abnormal segregated spermatozoa were observed in the epididymis of GTW-treated rats [17], [18]. However, the mechanism behind its anti-fertility effects remains unknown.

In this study, we focused on the effects of GTW on spermatogenesis and the potential mechanism behind it. During spermatogenesis, embryonic ectoderm development (EED) as target genes can bind a methyllysine histone [19], [20]. Therefore, EED is one of potential target for GTW binding. Our results revealed that GTW can affect male fertility by acting on spermatogenesis. *In vitro* and *in vivo* studies further revealed that this impairment in sperm formation starts as early as the SSC stage. *In silico* results suggest that GTW and methylated H3K9 compete to inhibit the receptor of EED. As GTW-induced infertility is transient and recoverable [15], our findings suggest that GTW may be a potential Chinese medicine for male contraception.

## Results

### GTW impairs spermatogenesis

To explore the possible impact of GTW on spermatogenesis and reproductive function, four groups of 4-week-old mice were administered intragastrically with GTW suspension at doses of 7.5 mg/kg/day (group 1), 15 mg/kg/day (group 2), 22.5 mg/kg/day (group 3) and 45 mg/kg/day (group 4). Group 5 is control. Pregnancy rates of all five groups, including control (group 5) were examined after treatment for 40 days. Pregnancy rates decreased with increasing doses of GTW treatment. At 45 mg/kg/day, the pregnancy rate even dropped to zero, which indicates a strong detrimental effect of GTW on fertility (data were shown by He *et al*. [21]).

**Figure 2:**
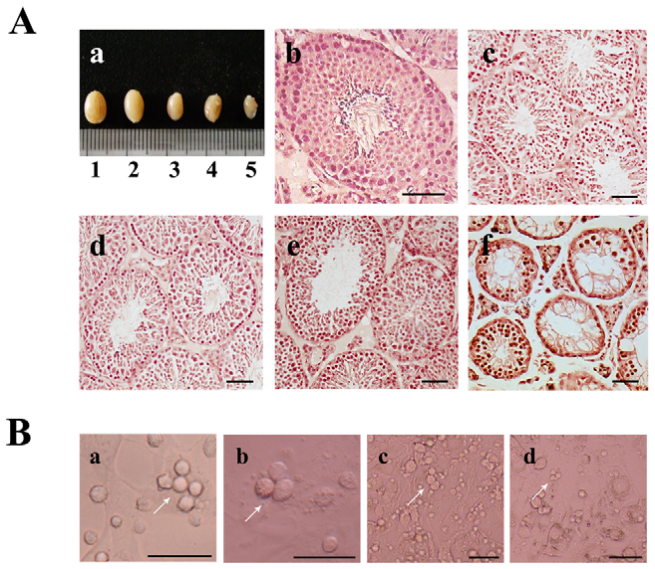
Macroscopic appearance and histology of testes (at 68 d of age) treated with GTW and morphology of SSCs cultured in medium with different doses of GTW. (A) Macroscopic appearance and morphology of testes treated with GTW or control. (a) Testis size. 1, control; 2. group 1; 3. group 2; 4, group 3; 5, group 4. (b–f) Histological morphology of GTW-treated or control testes. (b) Control; (c) group 1; (d) group 2; (e) group 3; (f) group 4. (B) Morphology of SSCs cultured in media with different doses of GTW or control. (a) Morphology of SSCs cultured for 12 h after isolation from 6-day-old mice. (b–d) Morphology of SSCs after 12 h treatment with control (b) or 10% (c) or 20% (d) GTW-containing serum. The scale bar represents 50 µm.

There were no significant changes in body size and weight of mice after treatment with low dose (7.5 mg/kg/day) GTW. However, the hair of the mice became withered and vertical after high dose (45 mg/kg/day) GTW treatment for 2 weeks. Control animals appeared normal. Testes of the treated mice were separated and examined morphologically. Size of testes decreased with increasing GTW dose (Figure 2A (a)). Histological analysis observed that the number of spermatozoa in the seminiferous tubules decreased, and this phenomenon was even more obvious in the high dose treatment (Figure 2A (b) – (f)). Many cavities were visible in group 4, and the arrangement of germ cells was disordered especially in group 4.

To analyze the influences of GTW *in vitro*, SSCs were isolated from 6-day-old mice. Morphological profiles of SSCs after cultivation for 12 h are shown (Figure 2B (a)). In accordance with previous studies [22], [23], serum containing GTW was added into the SSC medium at the concentration of 10% or 20%. SSCs were then cultured in these media for 12 h. After 12 h, some cell fragments began to appear, but most SSCs were relatively intact in morphology (Figure 2B (c) – (d)).

### GTW induces apoptosis in germ cells

**Figure 3:**
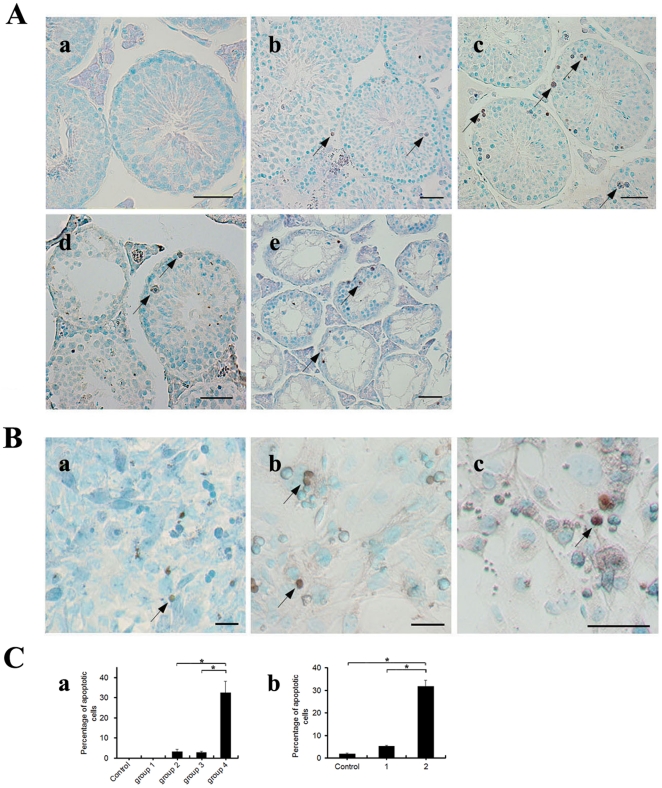
Apoptosis of male germ cells *in vivo* and *in vitro*. (A) Apoptosis in testes (at 68 d of age) from mice treated with GTW or control. (a) Control; (b) group 1; (c) group 2; (d) group 3; (e) group 4. Arrows indicate apoptotic germ cells. (B) Apoptosis of SSCs treated with GTW or control *in vitro*. (a–c) Apoptosis of SSCs after treatment for 12 h with control (a) or 10% (b) or 20% (c) GTW-containing serum. Arrows indicate apoptotic SSCs. (C-a) Percentage of apoptotic cells in total germ cells per field (Means ± SEM, n = 5), corresponding to the sections in (A). (C-b) Percentage of apoptotic cells in total SSCs per field (Means ± SEM, n = 5), corresponding to (B). *P<0.05. The scale bar represents 50 µm.

Using an *in situ* Apoptosis Detection Kit, apoptotic cells were stained brown with diaminobenzidine. There were few apoptotic cells in group 1, which was similar to the control group (Figure 3A (b)). However, in group 2, apoptotic cells significantly increased (Figure 3A (c)). When the dose of GTW was increased to 22.5 mg/kg/day (group 3), apoptosis-related cavities began to appear (Figure 3A (d)). For animals exposed to 45 mg/kg/day (group 4), although seminiferous tubules were pathologically atrophied and the majority of germ cells was depleted, there still were some cells that showed apoptotic traits among those that survived (Figure 3A (e)). We randomly chose 20 fields of view of each slide and calculated the ratio of apoptotic (Figure 3C (a)).

Noticeably, most of the apoptotic cells were distributed near basement membrane of the seminiferous tubules, where early stage germ cells including SSCs are located. Previous observations *in vitro* have demonstrated the great morphological impact of GTW on these cells. To find out if GTW induced SSC apoptosis in a direct manner, SSCs isolated from wild-type mice were cultured with serums containing different concentrations of GTW. The apoptosis rate of SSCs was analyzed after 12 h treatment (Figure 3B (a) – (c)). After 12 h, the number of apoptotic cells in the 10% GTW group was lower than in the 20% GTW group. However, they were both much higher than in the controls (Figure 3C (d)), which indicates an elevated level of GTW-induced apoptosis in SSCs.

### GTW down-regulates the dimethylation of H3K9 in germ cells

**Figure 4:**
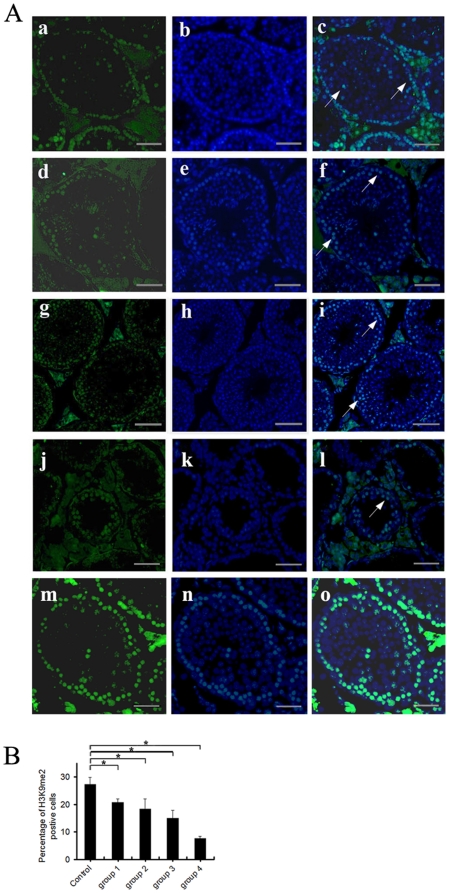
Histone epigenetic methylation of testes (at 68 d of age) with GTW. (A) Immunofluorescence of GTW treated testes with H3K9me2 antibody. (a, d, g, j, m) H3K9me2 staining of testis sections. (a) group 1; (d) group 2; (g) group 3; (j) group 4; (m) control. (b, e, h, k, n) DAPI staining. (b) group 1; (e) group 2; (h) group 3; (k) group 4; (n) control. (c, f, i, l, o) Corresponding merged images. Arrows indicate H3K9me2-positive germ cells. The scale bar represents 50 µm. (B) Percentage of H3K9me2-positive germ cells of the sections mentioned above (Means ± SEM, n = 5). *P<0.05.

To explore the temporal expression pattern of H3K9me2, testes of different ages in untreated mice were analyzed by immunofluorescence. Expression levels of dimethylated H3K9 was the highest in adult mice. However, it decreased gradually with increasing age, and there were few H3K9 positive cells in 360-day-old mice (Figure S1). Compared with controls, a few H3K9 dimethylated positive cells moved from the edge towards the lumen in groups 1–3 (7.5 mg/kg/day to 22.5 mg/kg/day), while there was no significant difference among the three groups (Figure 4B). When the dose was further increased, dimethylated cells decreased significantly (*P*<0.05). In group 4, although cells were almost destroyed, there were still a few scattered methylated positive cells in the tubules (Figure 4A).

**Figure 5:**
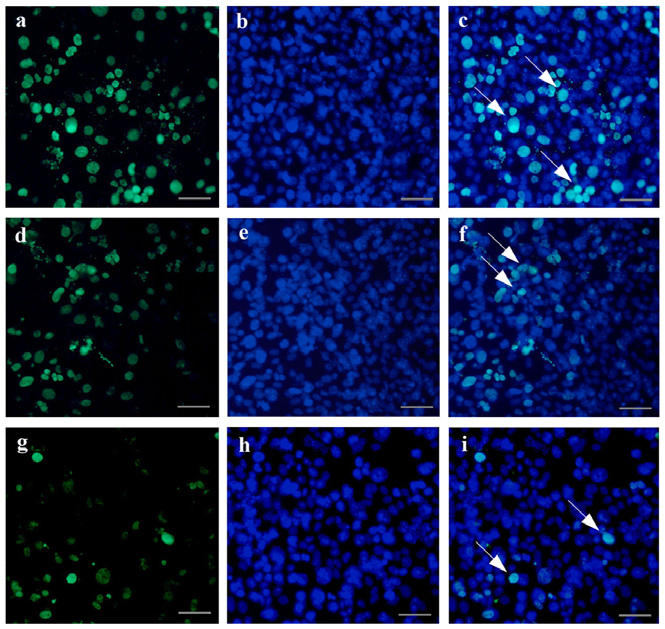
H3K9me2 expression in SSCs treated with GTW or control for 12 h. (a, d, g) H3K9me2 staining of SSCs treated with control (a), or 10% (d), or 20% (g) GTW-containing serum. (b, e, h) DAPI staining of SSCs treated with control (b), or 10% (e), or 20% (h) GTW-containing serum. (c, f, i) Corresponding merged images. Arrows indicate H3K9me2-positive SSCs. The scale bar represents 50 µm.

Similarly, we focused on the dimethylated state of H3K9me2 in SSCs. Gonocytes transformed into SSCs between 0 and 6 days postpartum [24], [25]. Protein from testes of 6-day-old mice was analyzed by western blotting. H3K9me2 was expressed in SSCs (Figure S2). Furthermore, SSCs were treated for 12 h with different concentrations of GTW or Triptolide, after which dimethylation levels of H3K9me2 were determined. The number of H3K9me2 positive cells in treated groups was lower than in the controls, and there was a significant difference between control and higher dosage groups (Figure 5, Figure S3, *P*<0.05), which is in agreement with the *in vivo* results.

### Inhibition mode of GTW

**Figure 6:**
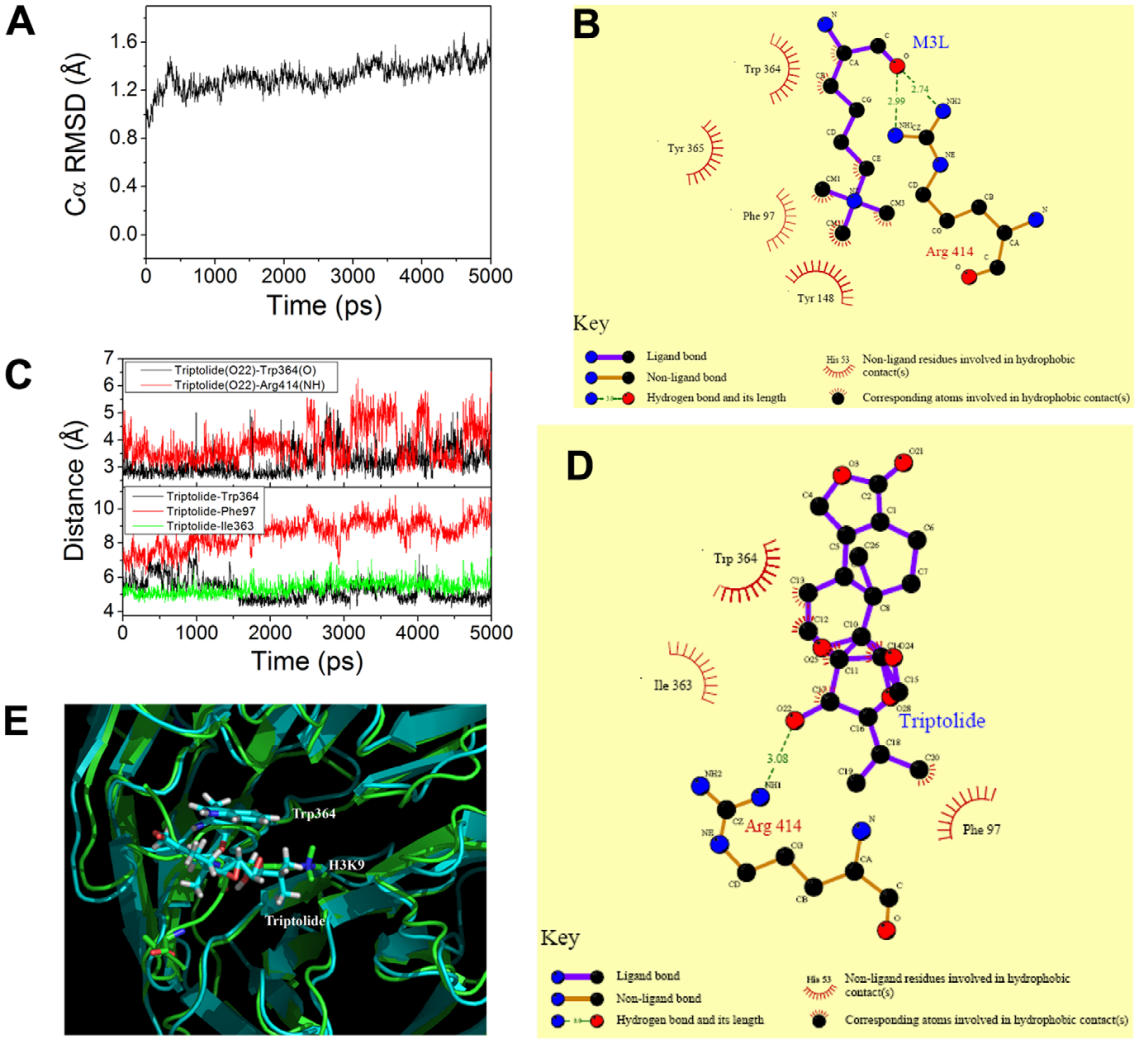
Molecular modeling, docking and MD simulation analysis of H3K9-EED and GTW-H3K9 interactions. (A), Simulation time vs. rmsd of the backbones of EED with GTW. (B) Two-dimensional representation for the interacting mode of H3K9 with EED, drawn by the LIGPLOT program. (C), Two-dimensional representation for the interacting mode of GTW with EED, drawn by the LIGPLOT program. (D), Simulation time vs. distance of hydrogen bond and hydrophobic contact for GTW. (E), Aligning of H3K9 (green) and GTW (cyan) in the EED hydrophobic pocket.

Triptolide is one of the primary active compounds in the GTW and was used to investigate the regulation mode. 5.0 ns molecular dynamics simulations were performed for the complex of triptolide and EED. To examine the variations in the intramolecular conformations of EED, the root-mean-squared deviation (rmsd) with respect to the initial structure was calculated. Simulation time versus rmsd of the backbone of the protein during the full simulation (5.0 ns of molecular dynamics) is presented in Figure 6A. The rmsd variations of EED model were about 1.3 Å. The results show that the complex became dynamically equilibrated after 3 ns of simulation.

The principal hydrophobic and hydrogen bonding interactions for H3K9-EED from the crystal structure and triptolide-EED from the nearest conformers to their average conformers were identified with Ligplot [26] and are represented in Figure 6B and 6C, respectively. For H3K9-EED, there were two important hydrogen bonds between the oxygen (O) atom in H3K9 and the nitrogen atom in residue Arg414(O…NH1), with a distance of 2.99 Å, and between the oxygen (O) atom in H3K9 and the nitrogen atom in residue Arg414 (O…NH2) with a distance of 2.74 Å. For triptolide-EED, there was one hydrogen bond between the oxygen (O22) atom in triptolide and the nitrogen atom in residue Arg414 (O…NH1), with a distance of 3.08 Å. Figure 6D (a) illustrates the variation of distance between O22 of GTW and NH1 of Arg414. The average distance of O22…NH1 (Arg414) is about 3.0 Å. In addition, there was another margin hydrogen bond between O22 of triptolide and oxygen atom in residue Trp364. This suggests that there are two hydrogen bonds for H3K9 and triptolide. Furthermore, there was a common hydrogen bond with Arg414 for methylated H3K9 and triptolide.

The hydrophobic contacts were defined by a distance of less than 6.5 Å between the hydrophobic center of EED and the ligand [27], [28]. The hydrophobic contacts for H3K9-EED and triptolide-EED are also shown in Figure 6B and 6C. Four hydrophobic interactions can be found for H3K9-EED: H3K9/Trp364, H3K9/Tyr365, H3K9/Tyr148, and H3K9/Phe97. For triptolide-EED, two stable hydrophobic contacts can be found in Figure 6D: triptolide/Trp364, and triptolide/Ile363. The hydrophobic contact of triptolide/Phe97 is rather weak. Comparing with H3K9, we found that there is also a common hydrophobic contact with Trp364 for H3K9 and triptolide.

Figure 6E illustrates the superposition of H3K9 and triptolide within an EED hydrophobic pocket. The dimethyl of triptolide and methylated H3K9 are mostly aligned well. It demonstrates that triptolide and H3K9 might have similar interaction mechanisms with EED. That is, Arg414 and Trp364 are key residues for triptolide and H3K9 effective binding.

## Discussion

Male infertility was found to be a side effect in the treatment of rheumatoid arthritis and psoriasis with GTW, sparking interest in GTW as a potential contraceptive drug. Although contraceptive drugs are generally used by women to prevent pregnancy, these drugs have many side effects on women's health. Therefore, the design of male contraception has become a new trend. Chinese traditional medicine is widely used for its gentle effect on health. GTW is one component of *Tripterygium Wilfordii* (other components include triptolide and triptolidenol [29]), and has been widely used clinically in recent years because of its low toxicity. In the 1980s, research indicated that GTW could affect the quantity and viability of spermatozoa. However, the effects were reversed after the termination of treatment. Thus, GTW could potentially be applied as a male contraceptive drug in the future.

Our histological results showed that high dosage treatment with GTW caused germ-cell-depleted cavities and degenerated tubules. TUNEL assays confirmed that GTW could induce SSCs apoptosis *in vivo* and *in vitro*. SSCs are the origin and source of mature germ cells, and SSC impairment would inevitably perturb the complete spermatogenetic process, causing a short term reduction in spermatozoa and ablated germ cells, though they can regenerate later.

Generally, functional degeneration in reproduction, such as high aberration yield, low numbers and low sperm vitality, are always associated with the mammalian aging process. Additionally, spermatogenesis could be controlled through epigenetic mechanisms such as histone modification (methylation, acetylation, phosphorylation, ubiquitination and sumoylation), which determine the activation of gene expression and the variation in chromatin structure during the germline cell development [30]. Abnormality in epigenetic state, including the methylation state of H3K9, might cause variations in spermatogenetic control or irregular development of germ cells, all of which could be potential factors in deteriorated fertility. We demonstrated that the methylation state of H3K9 was altered after GTW treatment. Additionally, triptolide and dimethylated or trimethylated H3K9 might have similar interaction mechanisms with EED detected by molecular dynamics simulation. Methylated H3K9 is stable after binding to EED protein. Because triptolide competes with methylated H3K9 for binding to EED, the methylated H3K9 might be dissociated, decreasing the level of methylated H3K9. Previous studies have demonstrated that H3K9me2 first appeared in Type B spermatogonia, and is expressed highly in pachytene spermatocytes [31]–[37]. But there are several reports showing that H3K9me2 is weak or is not expressed in pachytene spermatocyte [38]–[40]. Moreover, testes (composed of SSCs transformed from gonocytes) of 6-day-old mice were analyzed by western blotting, and immunofluorescence revealed that dimethylated H3K9 actually began to appear in a much earlier period, in Type A, or even in A_s_ cells (SSCs). Thus, one of inhibition mechanisms of spermatogenesis by GTW (including triptolide) treatment may be through alterations in the methylation of H3K9.

In summary, we studied the anti-fertility mechanism of GTW or triptolide. First, GTW can induce male germ cell apoptosis. Furthermore, altered dimethylation states of H3K9 may serve as another way to inhibit the process of spermatogenesis. We showed that methylation alterations might occur at early stages in SSCs. In addition, competitive binding to EED of triptolide was identified by molecular dynamics simulation. Therefore, GTW could be designed as a male contraceptive drug for clinical use in the future.

## Materials and Methods

### Animals

BALB/c mice and CD1 mice used in this study were purchased from SLAC Laboratory Animal Co. LTD, Shanghai China. Mice were housed in the specific pathogen free (SPF) facility at the Laboratory Animal Center, Shanghai University of Traditional Chinese Medicine. Four-week-old males were administered with GTW by gavage. Six-day-old males were used for the isolation of SSCs. All procedures involving animals were approved by the Institutional Animal Care and Use Committee of Shanghai, and were conducted in accordance with the National Research Council Guide for Care and Use of Laboratory Animals [SYXK (Shanghai 2007-0025)].

### Intragastric administration

GTW was purchased from Shanghai Fudan Fuhua Pharmaceutical Industry Company Limited. Four-week-old BALB/c male mice were randomly divided into 5 groups of 10 animals in each. In groups 1–4, GTW was given by gastric gavage for 40 days. Group 1 received GTW 7.5 mg/kg/day; group 2 received GTW 15 mg/kg/day; group 3 received GTW 22.5 mg/kg/day; group 4 received GTW 45 mg/kg/day; group 5 was fed with distilled water instead of GTW for control.

### Preparation of mediated serum of GTW

Wild-type BALB/c male mice were administered with 45 mg/kg/day GTW or 17 µg/kg Triptolide [41] for 3 days. Two hours after the intragastric administration, blood was collected and centrifuged at 13,400×*g* for 15 min. Supernatant was precipitated at 4°C overnight and the serum was carefully moved to a new Eppendorf tube. Later the concentration of Tripterygium wilfordii and Triptolide in the serum was dected with spectrophotomer. After filtering for sterilization, the serum was stored at −20°C for further study.

### Histological examination

Testes from mice were fixed with 4% paraformaldehyde (PFA) and 0.2% glutaraldehyde in PBS overnight at 4°C. After being dehydrated through 50%, 70%, 85%, 95% and 100% ethanol, the testes were vitrificated in xylene and later embedded in paraffin. Sections of 6 µm thickness were mounted on slides, de-waxed with xylene and stained with haematoxylin for histological analysis. Pictures were taken using a Nikon Eclipse E600 microscope equipped with a Nikon DXM 1200 digital camera (Tokyo, Japan).

### Immunofluorescence

For immunohistochemistry, tissue sections were deparaffinized, equilibrated in phosphate-buffered saline (PBS) for 10 min, digested by 0.05% trypsin at 37°C for 15 min, and then washed with PBS and blocked in 10% normal goat serum at room temperature for 10 min. Slides were subsequently incubated with primary antibodies at 4°C overnight (mouse anti-H3K9me2; 1∶100; Abcam). Sections were washed with PBS before incubation with fluorescein isothiocyanate (FITC) conjugated secondary antibody (goat anti-mouse immunoglobulin G; 1∶150; Invitrogen) for 30 min at 37°C in darkness to detect H3K9me2. Sections were then washed with PBS, and incubated in DAPI (1∶10000, Sigma) for 18 min at 37°C in darkness.

After treatment with serum containing GTW, SSCs were fixed with 4% PFA in PBS for 20 min at room temperature, washed with PBS and incubated in 3% H_2_O_2_ for 15 min at room temperature. Finally, SSCs were blocked with goat serum and incubated with mAb of mouse anti-H3K9me2 (1∶100) and polyantibody of rabbit anti-PLZF (1∶50) for 1 h, washed by PBS, then incubated with secondary antibody (goat anti-mouse IgG conjugated TRITC, 1∶150; goat anti-rabbit IgG conjugated FITC, 1∶150, Abcam) for 30 min at 37°C in darkness, stained with DAPI (1∶10000) for 20 min, obscured under the fluorescence microscopic after washing by PBS three times.

### Isolation, culture and GTW treatment of spermatogonial stem cells (SSCs)

CD1 mice at 6 days of age were used as donor mice for establishing SSC cultures. The two-step enzymatic digestion of Nagano *et al*. and Wu *et al.* was used [42]–[48]. Seminiferous tubules were collected from decapsulated testes and dissociated enzymatically in D-Hanks buffer containing 1 mg/ml collagenase (type IV, Sigma) at 37°C for 15 min. Then the testicular tissue was digested by 0.05% trypsin prepared with D-Hanks containing 1 mM EDTA at 37°C for 10 min. After neutralization by DMEM with 10% fetal bovine serum (FBS), lysates were centrifuged at 300×*g* for 5 min, the precipitated cells were mixed with BD™ IMaganti-mouse CD 90.2 (Thy-1.2) particles and purified according to instructions of the kit. Finally, the isolated cells were transferred onto STO cell feeder layers (mouse SIM embryonic fibroblasts, strain SIM, 5×10^4^ cells/cm^2^, ATCC). The composition of germ cells medium was as follows: minimum essential medium α medium (MEM-α) supplemented with 15% FBS, 1 mM sodium pyruvate, 1 mM non-essential amino acids, 2 mM L-glutamine, 0.1 mM β-mercaptoethanol (Sigma), 10 ng/ml LIF (Santa Cruz Biotechnology) and 15 mg/l penicillin. SSCs were cultivated at 37°C for 12 h under 5% CO_2_, and then treated with different doses of GTW or Triptolide for 12 h.

### Western blot analyses

For protein analyses, testes were isolated from 6-day-old or adult mice and homogenized on ice with lysis buffer containing protease and phosphatase inhibitors (1% Triton X-100, 50 mM Tris–HCl, 5 mM EDTA, 1% aprotin and 1% leupeptin). The cell lysates were centrifuged (13400 g/min for 30 min), and total protein was qualified by the BCA Protein Assay Kit (Pierce, Rockford, IL), then separated by 8% SDS-PAGE and transferred to nitrocellulose membrane. The expression of H3K9me2 was probed by western blotting with mouse anti-H3K9me2 primary antibody (1∶100; Chemicon). Secondary detection included incubation with HRP conjugated goat anti-mouse IgG antibody (1∶20,000; Amersham Pharmacia Biotech). Signals were detected using ECL western blotting Kit (Amersham Pharmacia Biotech).

### TUNEL analysis of cell apoptosis

The samples were analyzed by terminal deoxynucleotidyl transferase-mediated dUTP nick end labeling (TUNEL) assay using *in situ* Apoptosis Detection Kit (TaKaRa, Japan) according to the instructions.

### Molecular modeling and docking

Molecular modeling was used to investigate the mechanism behind the decrease in methylated levels of H3K9 by triptolide. The complex of EED (embryonic ectoderm development) and methylated H3K9 was extracted from Brookhaven Protein Databank (PDB code: 3K27) [49]. The structure of triptolide is shown in Figure 1. The structure was optimized using Tripos force field. All calculations were performed on the in-house Xeon (1.86GHz) cluster.

The active site was determined based on the co-crystallized H3K9/EED complex. First, the methylated H3K9 was extracted from the EED complex, and then the remaining EED structure was completed by adding the missing polar hydrogen atoms. This structure was optimized with constrained molecular dynamics. Partial atom charges of EED were calculated with Kollman-all-atom approximation [50]. Gasteiger-Hückel charges were calculated for tripterygium glycoside, as recommended in the AutoDock 3.0 package, which was used for performing automated docking of inhibitors to EED. The development and the principle of this software have been described elsewhere [51].

### Molecular Dynamics Simulations

MD simulations and energy minimizations were performed using the AMBER8.0 simulation package [52] and the parm99 force field [53] with the TIP3P water model [54] The initial coordinates of the complex for triptolide and EED were extracted from Autodock. Hydrogen atoms were added using the LEAP module of AMBER8.0. Antechamber [55] was used to handle the force field of triptolide. Ten Cl ions were placed around the complex to maintain the system's neutrality. The SHAKE algorithm [56] was used to constrain bonds involving hydrogen atoms. The complex was solvated in a rectangular box of water, with the shortest distance between any protein atom and the edge of the box approximately 10 Å. Particle Mesh Ewald (PME) [57] was employed to calculate long-range electrostatic interactions. Then the complex was minimized using the PMEMD module of AMBER8.0. This minimization consisted of 1000 steps with the steepest descent method. The systems were equilibrated at 298 K for 20 ps. After 20 ps equilibration, MD simulation was employed to record time trajectory. The complex was simulated for 5.0 ns at 298 K. The time step used in all calculations was 2.0 fs. Coordinates were saved every 1 ps for the purpose of subsequent analysis.

### Statistical Analysis

All data are expressed as the mean ± standard error of the mean (SEM). Results were analyzed by Chi-square test. Data between groups were compared using partition analysis. *P*-values less than 0.05 (*P*<0.05) were considered significant.

## Supporting Information

Figure S1 **Immunoflourescence of testes.** Immunofluorescence of testes of different ages with H3K9me2 antibody. (a, d, g, j, m) H3K9me2 staining on 10-day (a), 20-day (d), adult (g), 120-day (j), 360-day (m) old testes. (b, e, h, k, n) DAPI staining on 10-day (b), 20-day (e), adult (h), 120-day (k), 360-day (n) old testes. (c, f, i, l, o) Corresponding merged images. Arrows indicate H3K9me2-positive germ cells. The scale bar represents 50 µm.
(TIF)

Figure S2 **Western blotting analysis of protein obtained from adult testes or 6-day-old testes, probed with mouse anti-H3K9me2 antibody.** The expression of β-tubulin was used as an internal standard for normalization.
(TIF)

Figure S3 **H3K9me2 expression level on SSCs analyzed with immunofluorescence.** Three lines respectively indicated as 10% FBS, 10% serum containing Triptolide and 20% serum containing Triptolide. (a, d, g) DAPI stained. (b, e, h) stained with polyantibody for PLZF. (c, f, i) immunolabeled with mAb against H3K9me2, scale bar represents 50 µm.
(TIF)
