# Supplementary figures and images for: Male Germ Cell Apoptosis and Epigenetic Histone Modification Induced by Tripterygium wilfordii Hook F
Source: PLoS One. 2011 Jun 15;6(6):e20751. doi: 10.1371/journal.pone.0020751 (PMC3115959; doi:10.1371/journal.pone.0020751)

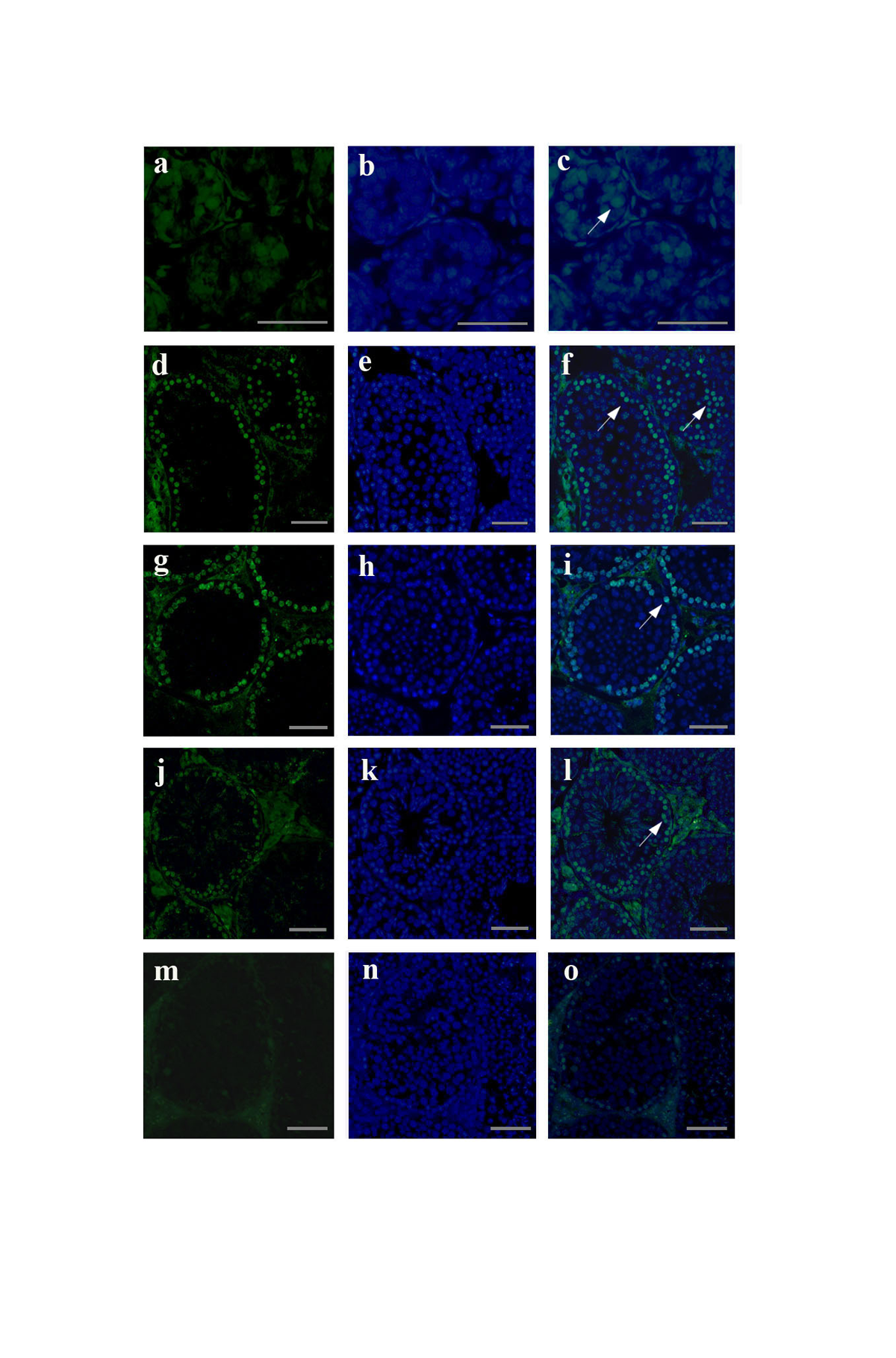

Supplement: Figure S1 — Immunoflourescence of testes. Immunofluorescence of testes of different ages with H3K9me2 antibody. (a, d, g, j, m) H3K9me2 staining on 10-day (a), 20-day (d), adult (g), 120-day (j), 360-day (m) old testes. (b, e, h, k, n) DAPI staining on 10-day (b), 20-day (e), adult (h), 120-day (k), 360-day (n) old testes. (c, f, i, l, o) Corresponding merged images. Arrows indicate H3K9me2-positive germ cells. The scale bar represents 50 µm. (TIF) [file pone.0020751.s001.tif]

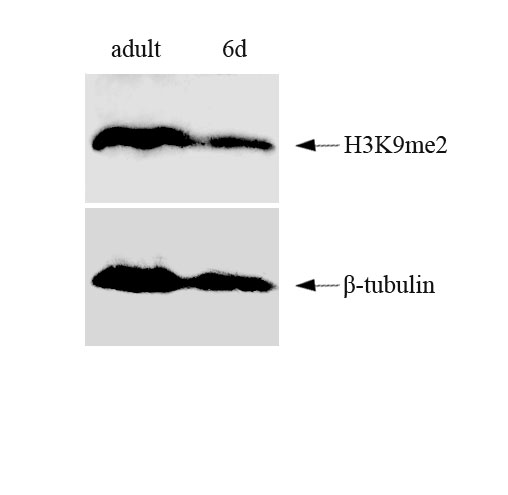

Supplement: Figure S2 — Western blotting analysis of protein obtained from adult testes or 6-day-old testes, probed with mouse anti-H3K9me2 antibody. The expression of β-tubulin was used as an internal standard for normalization. (TIF) [file pone.0020751.s002.tif]

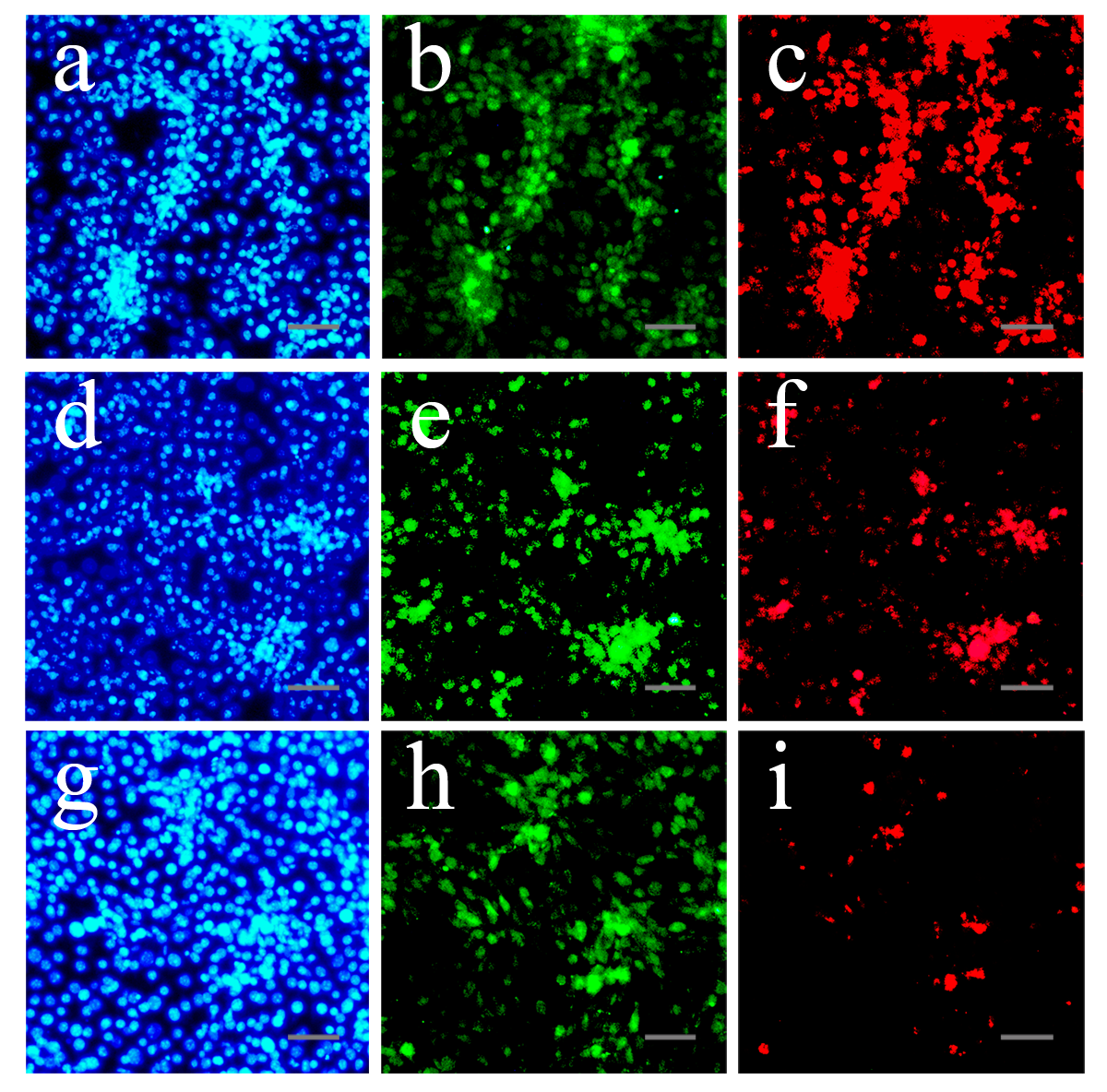

Supplement: Figure S3 — H3K9me2 expression level on SSCs analyzed with immunofluorescence. Three lines respectively indicated as 10% FBS, 10% serum containing Triptolide and 20% serum containing Triptolide. (a, d, g) DAPI stained. (b, e, h) stained with polyantibody for PLZF. (c, f, i) immunolabeled with mAb against H3K9me2, scale bar represents 50 µm. (TIF) [file pone.0020751.s003.tif]
